# A CBCT Morphometric Study of Hyoid Bone According to Skeletal and Breathing Patterns Using Multi-Factor Robust ANOVA

**DOI:** 10.3390/healthcare13192423

**Published:** 2025-09-24

**Authors:** Busra Ozturk, Guldane Magat, Mucahid Yildirim, Alparslan Esen

**Affiliations:** 1 Department of Oral and Maxillofacial Radiology, Faculty of Dentistry, Necmettin Erbakan University, 42090 Konya, Türkiye; gul_dent@hotmail.com; 2 Department of Orthodontics, Faculty of Dentistry, Necmettin Erbakan University, 42090 Konya, Türkiye; mucahidyildirim@erbakan.edu.tr; 3 Department of Oral and Maxillofacial Surgery, Faculty of Dentistry, Necmettin Erbakan University, 42090 Konya, Türkiye; aesen@erbakan.edu.tr

**Keywords:** angle’s classification, cone beam tomography, breathing, hyoid bone, ANOVA

## Abstract

**Background/Objectives**: The hyoid bone plays a central role in functions such as swallowing, speech, and airway maintenance, and its morphology may vary with anatomical and functional parameters. This study aimed to evaluate the influence of skeletal class, respiratory mode, age, and sex on the morphometric features of the hyoid bone using cone-beam computed tomography (CBCT). **Methods**: A total of 560 CBCT scans (295 females, 265 males; aged 8–73 years) were retrospectively analyzed. Hyoid angle, horizontal length, and vertical height were measured using Dolphin 3D software. Participants were categorized by skeletal class (I, II, III), breathing pattern (nasal vs. oral), and age group. Data were analyzed using robust three-way ANOVA and Bonferroni post hoc tests. **Results**: In females, nasal breathers exhibited significantly larger hyoid angles and vertical heights than oral breathers (*p* < 0.001), independent of age and skeletal class. In males, both age and breathing mode significantly influenced hyoid angle and vertical length (*p* < 0.001). Vertical height was also significantly greater in skeletal Class I compared to Class III (*p* = 0.008). Notably, significant respiration–skeletal class interaction was found in females (*p* = 0.029) but not in males. **Conclusions**: Hyoid bone morphology is affected by age, breathing pattern, and skeletal class, with sex-specific differences. Nasal breathing and younger age were associated with more inferior and angularly favorable hyoid positions, which may have implications for airway stability and craniofacial development.

## 1. Introduction

The hyoid bone stands out in the craniofacial skeleton as the only anatomic structure that remains unattached to neighboring bones, maintaining its position through muscular and ligamentous support. With its U-shaped or horseshoe-like configuration, the hyoid rests in the front of the neck, positioned between the lower jaw and the larynx, roughly aligning with the third cervical vertebra [1]. Positioned just above the thyroid cartilage, the hyoid is suspended within a complex system of muscles and connective tissues rather than being anchored to adjacent bones [2].

Structurally, the hyoid consists of three main components: a central segment known as the body, and two projections—greater horns (cornu majus) and lesser horns (cornu minus) [3]. Developmentally, the upper portion of the hyoid body together with the lesser horns originates from the second pharyngeal arch, whereas the lower body region and the greater horns arise from the third pharyngeal arch [4]. Through multiple muscular connections, the hyoid maintains functional and structural associations with the skull base, mandible, tongue, scapula, sternum, pharynx, and thyroid cartilage [5].

Since the hyoid bone lacks direct bony connections, its spatial position is maintained exclusively by muscular forces. This position is influenced by several factors, including the elastic properties of the laryngeal and tracheal membranes, gravitational effects on the larynx, and the activity and length of surrounding muscles. Superiorly, muscles link the hyoid to the mandible and cranial structures, while inferior connections extend to the scapula, thyroid cartilage, and manubrium sterni. These muscular attachments regulate both the movement and stabilization of the hyoid and larynx [6]. Based on their location, these muscles are grouped as suprahyoid or infrahyoid. Located above the hyoid, the suprahyoid muscle group includes the digastric, stylohyoid, mylohyoid, and geniohyoid muscles, all of which work collectively to lift the hyoid bone during functions such as swallowing. Situated beneath the hyoid, the infrahyoid muscles—namely sternohyoid, sternothyroid, omohyoid, and thyrohyoid—primarily act to lower the bone, assisting in coordinated neck and swallowing movements [7].

Functionally, the hyoid bone is integral to essential orofacial activities such as maintaining cranial and cervical posture, ensuring airway patency, supporting tongue function, and contributing to phonation via its association with the larynx [8]. In addition, the hyoid contributes to key processes such as swallowing, chewing, breathing, and safeguarding against the backward flow of ingested material [9]. The anatomical position of the hyoid can be affected by head posture, sex differences, jaw relationships, and craniofacial growth patterns.

Previous studies have demonstrated correlations between hyoid positioning and mandibular position, with subsequent implications for airway configuration [10,11,12,13]. In forensic and anthropological studies, variations in hyoid shape and structure have been utilized as indicators for determining an individual’s sex and estimating age [14,15,16]. Additionally, an individual’s predominant breathing mode (oral vs. nasal) has been suggested to influence hyoid location [17,18,19,20,21].

Although numerous studies have investigated the relationship between hyoid bone position and skeletal or respiratory characteristics, the findings remain inconclusive. Variations in methodology, population, and measurement parameters have led to conflicting results regarding the role of age, sex, skeletal class, and breathing pattern. Furthermore, many studies have relied on two-dimensional cephalometry or limited subgroup analyses, which may not fully capture the complex interplay of factors influencing hyoid morphology [22,23].

Given this gap, the present study aims to comprehensively evaluate the horizontal and vertical positions of the hyoid bone, as well as its angular relationships, in association with skeletal and breathing characteristics using cone-beam computed tomography (CBCT) imaging. Uniquely, this study applies a three-way robust ANOVA model to simultaneously analyze the effects and interactions of age, sex, skeletal class, and breathing mode—offering a statistically rigorous and multifactorial approach rarely used in the field. We hypothesize that hyoid morphology differs significantly by breathing pattern, skeletal classification, and age group, with sex-specific patterns, and that a multivariable model will reveal interactions not captured in single-variable studies.

## 2. Materials and Methods

### 2.1. Ethical Approval

Ethics committee approval for this retrospective study was obtained by Necmettin Erbakan University Faculty of Dentistry, Faculty of Dentistry, Drug and Non-Medical Device Research Ethics Committee on 25 May 2023 with protocol number 2023/299, and the scientific and ethical appropriateness of the study was approved.

### 2.2. Study Population and Power Calculation

CBCT images obtained for diagnostic purposes of patients who applied to Necmettin Erbakan University Faculty of Dentistry, Department of Oral, Dental and Maxillofacial Radiology between February 2019 and October 2024 with various dental complaints were retrospectively analyzed.

Before starting the study, a power analysis was performed by ANOVA method using the G*Power v3.1.9.7 program to determine the number of individuals to be included in the groups. According to the 95% confidence level (1 − α), 95% test power (1 − β), Cohen’s f = 0.303 effect size and multi-way analysis of variance parameters, it was determined that a minimum of 240 individuals should be included in the study, with at least 4 cases (209/60 ≅ 4) in each group. In order to increase the statistical power of the study, it was decided to include more records than the minimum number of cases initially determined.

### 2.3. Inclusion Criteria

The study included patients aged ≥8 years with no congenital or acquired craniofacial anomalies, history of craniofacial trauma or surgery, cleft lip/palate, systemic syndromes, or missing teeth affecting occlusion, and with CBCT images of optimal diagnostic quality allowing clear identification of reference points.

### 2.4. Exclusion Criteria

Patients were excluded if they had congenital or acquired craniofacial anomalies, a history of craniofacial trauma or surgery, cleft lip/palate, systemic syndromes, missing teeth affecting occlusion, suboptimal CBCT image quality, or unclear reference points.

### 2.5. CBCT Acquisition Protocol

In this retrospective study, CBCT images of 560 male and female patients aged 8–73 years with high diagnostic quality who met the inclusion criteria were analyzed. The CBCT images used in the study were obtained with Morita 3D Accuitomo 170 and NewTom GiANO 3D devices. The Morita 3D Accuitomo 170 (J Morita MFG Corp., Kyoto, Japan) was operated at 90 kVp and 5 mA, with an irradiation time of 17.5 s, 0.25 mm voxel size, 140 mm × 100 mm FOV, 360° data acquisition and no additional filtering. The NewTom GiANO 3D device (Verona, Italy) was operated at 90 kVp and 10 mA, with an irradiation time of 18 s, 0.15 mm voxel size, 140 mm × 100 mm FOV, 360° data acquisition and no additional filtering. Both devices were calibrated before each patient and radiographs were taken by the same technician according to standard protocols.

### 2.6. Measurement Procedure

The included CBCT images were reviewed by a single observer with three years of experience in oral diagnosis and maxillofacial radiology (BO). Image data were saved in DICOM (Digital Imaging and Communications in Medicine) format and measurements were performed using 3D Dolphin 11.95 Imaging software (Dolphin Imaging & Management Solutions^®^, Chatsworth, CA, USA). Mark points and reference planes were checked in axial, coronal and sagittal planes, and precise measurements were made on three-dimensional models. The observer was blinded to participants’ skeletal classification and breathing pattern to minimize measurement bias.

### 2.7. Three-Dimensional Cephalometric Measurements

**Figure 1:**
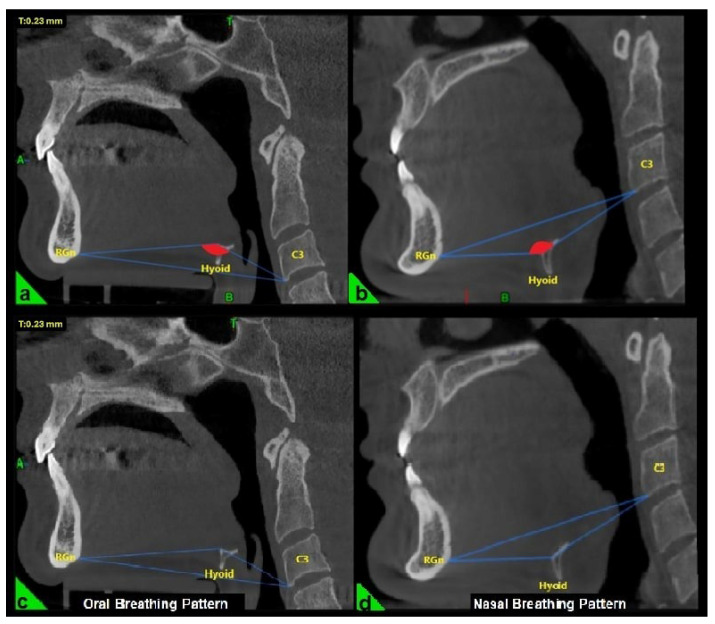
Evaluation of the hyoid angle in three-dimensional cephalometric analysis. RGn: Most posterior-lower point of the mandibular symphysis; Hyoid: Center of the hyoid bone; C3: Anterior-lower point of the third vertebra of the cervical vertebra. (**a**) Negative hyoid angle (**b**) Positive hyoid angle (**c**) Oral breathing pattern due to negative hyoid angle (**d**) Nasal breathing pattern due to positive hyoid angle.

In the three-dimensional cephalometric analysis, three key measurements were performed to evaluate the position and morphology of the hyoid bone. The hyoid angle was defined as the angle formed by two lines: one extending from the anterior-inferior point of the third cervical vertebra (C3) to the most anterior point of the hyoid bone, and the second from this point to the retrognathion (RGn), which is the most posterior-inferior point of the mandibular symphysis (Figure 1a,b) [22].

**Figure 2:**
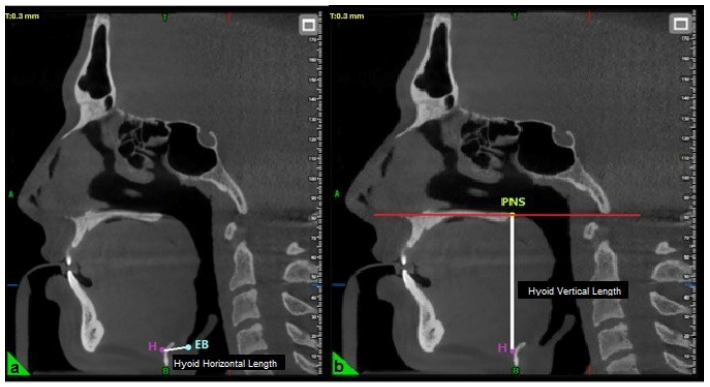
Evaluation of hyoid horizontal (**a**) and vertical length (**b**) in three-dimensional cephalometric analysis. H: Top point of the hyoid bone; EB: The lowest point of the posterior surface of the epiglottis; PNS: Posterior nasal spina.

The horizontal length of the hyoid bone was measured as the linear distance between point H (the superior point of the hyoid bone) and point EB (the most inferior point on the posterior surface of the epiglottis) (Figure 2) [23]. The vertical length was determined by measuring the perpendicular distance from the palatal plane (PL) to point H (Figure 2) [19].

### 2.8. Participant Grouping Criteria

Sex-based categorization was applied in this study. A total of 560 CBCT scans were analyzed, comprising 295 female and 265 male patients. Participants ranged in age from 8 to 73 years and were stratified into five age groups for subgroup analysis: 8–18 years, 19–29 years, 30–40 years, 41–51 years, and over 52 years.

Skeletal classification was determined by calculating the ANB angle on sagittal sections of the CBCT images using Dolphin 3D software (Dolphin Imaging & Management Solutions^®^, Chatsworth, CA, USA), based on the principles of Steiner analysis [24]. The ANB angle was obtained by subtracting the SNB (sella–nasion–point B) angle from the SNA (sella–nasion–point A) angle, which reflects the anteroposterior relationship of the maxilla and mandible to the cranial base. The Sella (S) cephalometric point used in this measurement is the geometric midpoint of the sella turcica located on the sphenoid bone. The nasion (N) cephalometric point is defined as the most anterior intersection of the nasofrontal suture with the sagittal plane and the deepest point of the recess in that region. Point A (Subspinal point) (A) is the deepest point in the midsagittal plane of the bony concavity located between the anterior nasal spine and the prosthion, while point B (Supramental point) (B) is the deepest point of the bony concavity located between the infradental and pogonion of the mandibular symphysis [25]. All measurements were performed on mid-sagittal slices aligned with standard cephalometric reference planes. Based on the measured ANB values, patients were categorized into three skeletal classes: Class I (ANB angle between 0° and 4°), Class II (ANB > 4°), and Class III (ANB < 0°).

The breathing pattern of each participant was classified as either nasal or oral based on the “hyoid triangle” technique, applied to sagittal CBCT sections using Dolphin 3D software (Dolphin Imaging & Management Solutions^®^, Chatsworth, CA, USA). Only this method was used to determine the breathing pattern. This method involves constructing a triangle using three anatomical landmarks: the anterior-inferior point of the third cervical vertebra (C3), the most anterior point of the hyoid bone, and the retrognathion (RGn). In this configuration, the RGn–C3 line serves as the base, and a triangle is formed by connecting these three points. When the hyoid bone is positioned above the RGn–C3 plane, the triangle has a superior vertex and is considered a “negative triangle,” indicating an oral breathing pattern (Figure 1a,c). Conversely, when the hyoid lies below the RGn–C3 line, the triangle takes on a “positive” configuration, which is indicative of nasal breathing (Figure 1b,d) [22].

To assess measurement reliability, 50 CBCT scans were randomly selected for both intra- and inter-observer evaluations. Intra-observer reliability was assessed by having the primary examiner (BO), with three years of experience in oral and maxillofacial radiology, repeat all measurements after a two-week interval. For inter-observer reliability analysis, a second examiner (GM), with sixteen years of experience in the same field, independently performed the same measurements on the selected scans. Both observers followed identical measurement protocols. Intraclass correlation coefficients (ICC) were calculated for both assessments, and values above 0.90 were considered indicative of excellent agreement [26].

### 2.9. Statistical Analysis

Data were analyzed using the JAMOVI V2.3.22 program. Compliance with normal distribution was evaluated by Shapiro–Wilk test. For the comparison of non-normally distributed data according to age, respiratory and skeletal pattern groups, three-way Robust ANOVA was applied using the Walrus package. Multiple comparisons were performed with the Bonferroni test. Analysis results are reported as pruned mean ± standard error of the mean for quantitative data. Statistical significance level was accepted as *p* < 0.05.

## 3. Results

The average age of the 560 participants was 33.74 ± 14.96 years. When the sex distribution is analyzed, 52.7% of the participants were female (295 people) and 47.3% were male (265 people), and in terms of breathing pattern, 28.6% were oral (160 people) and 71.4% were nasal (400 people) (Table 1).

In Table 2, the morphometric measurements of the participants are presented in detail with descriptive statistics. The mean hyoid angle was 88.79° ± 130.86°, hyoid horizontal length 14.08 mm ± 3.08 mm and hyoid vertical length 62.87 mm ± 8.14 mm.

Among female participants, hyoid angle values demonstrated a statistically significant difference based on breathing pattern (*p* < 0.001), independent of age or skeletal pattern classification. No significant effects were observed for age group (*p* = 0.32), skeletal pattern (*p* = 0.998), or the two-way interactions of age and breathing (*p* = 0.605), age and skeletal pattern (*p* = 0.600), or breathing and skeletal pattern (*p* = 0.319). Additionally, the three-way interaction did not reach statistical significance (*p* = 0.088). In contrast, male participants exhibited a significant effect of age group on hyoid angle (*p* = 0.011), with post hoc comparisons revealing notable differences particularly between the 19–29 and 41–51 age categories. Breathing pattern also had a significant effect (*p* < 0.001), as did the interaction between age and breathing pattern (*p* = 0.002), indicating consistent differences between oral and nasal breathers across age groups. However, skeletal pattern (*p* = 0.377), age–skeletal interaction (*p* = 0.359), and breathing–skeletal interaction (*p* = 0.676) was not statistically significant. Notably, a significant three-way interaction among age, breathing pattern, and skeletal classification was observed in males (*p* = 0.029) (Table 3).

Among female participants, hyoid horizontal length varied significantly across age groups (*p* = 0.009), regardless of breathing pattern or skeletal classification. Post hoc comparisons indicated that this difference was particularly pronounced between the 8–18 age group and both the 30–40 and over 52 age groups. No statistically significant differences were observed in relation to breathing pattern (*p* = 0.920), skeletal pattern (*p* = 0.940), or any two-way interactions, including age–breathing (*p* = 0.709), age–skeletal (*p* = 0.175), and breathing–skeletal (*p* = 0.369) combinations. The three-way interaction was also not significant (*p* = 0.290). In male participants, no significant differences were found in hyoid horizontal length across any of the examined variables or interactions. Specifically, there were no statistically significant effects of age group (*p* = 0.170), breathing pattern (*p* = 0.170), or skeletal classification (*p* = 0.588), nor were there any significant two-way or three-way interactions (all *p* > 0.05) (Table 4).

In female participants, hyoid vertical height differed significantly among age groups (*p* < 0.001), with post hoc analyses revealing marked differences between the 8–18 and 19–29 age groups compared to the older cohorts. Breathing pattern also had a significant effect on hyoid vertical height (*p* < 0.001). Additionally, a significant interaction between breathing pattern and skeletal classification was found (*p* = 0.029), indicating notable differences in vertical height between oral and nasal breathers within each skeletal class. No significant differences were observed for skeletal classification alone (*p* = 0.947), nor for the age–breathing (*p* = 0.232), age–skeletal (*p* = 0.388), or three-way interaction (*p* = 0.122). Among male participants, hyoid vertical height also varied significantly across age groups (*p* < 0.001), particularly between the 8–18 age group and all older groups, as well as between the 19–29 and ≥52 age groups. Breathing pattern was again significantly associated with hyoid vertical height (*p* < 0.001). Moreover, skeletal classification showed a significant effect (*p* = 0.008), with a notable difference identified between Class I and Class III. No statistically significant effects were found for age–breathing (*p* = 0.109), age–skeletal (*p* = 0.127), breathing–skeletal (*p* = 0.536), or three-way interaction (*p* = 0.373) terms (Table 5).

## 4. Discussion

This study comprehensively assessed hyoid bone morphology—including angular, horizontal, and vertical parameters—across skeletal classifications, age groups, sex, and breathing patterns using CBCT imaging. Key findings demonstrated that age and breathing pattern exerted significant effects on hyoid position, while skeletal pattern influenced specific metrics, particularly in males. These results reaffirm the dynamic nature of the hyoid bone, which—unlike other craniofacial bones—does not articulate directly with any other bone and is thus sensitive to functional and postural alterations in surrounding musculature and airway anatomy [2,7].

While the literature remains inconsistent regarding sex-based differences in hyoid position—some reporting no differences [8,27,28,29], others indicating a more anterior-inferior placement in males [12,30,31,32]—this study found age-related increases in vertical hyoid distance in both sexes, suggesting inferior displacement over time. Notably, in females, horizontal distance increased significantly with age, especially between younger (8–18) and older (>30) cohorts. These trends mirror longitudinal findings by Kollias and Krogstad [31], who observed continued craniofacial growth and inferior hyoid displacement into adulthood, who observed continued craniofacial growth and inferior hyoid displacement into adulthood.

Skeletal classification did not consistently emerge as a dominant determinant of hyoid position across all parameters. While some studies found retruded hyoid positions in Class II patterns [12], others—including Kocakara et al. [11]—reported no significant association when respiratory mode was controlled. In this study, skeletal class did not significantly affect horizontal metrics; however, in males, vertical hyoid height decreased from Class I to Class III, suggesting a more superior placement in more prognathic skeletal types. These results align with findings by Adamidis and Spyropoulos [33], who found significant differences in hyoid position and angles between Class I and Class III malocclusions, with Class III subjects—particularly males—showing more anterior positioning and reduced hyoid angles. Tallgren and Solow [7] demonstrated that hyoid position is influenced by mandibular position, cervical inclination, and craniocervical angulation. In our findings, skeletal pattern did not significantly affect horizontal distances in either sex. However, in males, vertical distance decreased from Class I to Class III, indicating a more superior hyoid placement in advanced skeletal classes.

Hyoid bone position is influenced not only by anatomical structures but also by functional factors such as breathing type. Prior studies have yielded contradictory findings: some observed a superior hyoid in oral breathers [21,34], while others reported an inferior displacement [17,18]. In the present study, nasal breathers—both male and female—exhibited greater vertical hyoid distances (inferior placement), whereas oral breathers had shorter distances (superior placement). Among females, the hyoid angle was significantly influenced only by breathing pattern, with nasal breathing associated with more positive angles and oral breathing with more negative angles. Among males, the hyoid angle was influenced by both age and breathing type. These results indicate that functional factors, particularly breathing patterns, may exert a greater influence on hyoid morphology than skeletal factors. When age, breathing pattern, and skeletal pattern were analyzed jointly, no significant combined effects were found for females. In contrast, in males, these three variables significantly influenced hyoid angle, highlighting the multifactorial determinants of hyoid positioning. From a clinical standpoint, changes in hyoid angle may impact upper airway patency and respiratory function and thus should be considered in individualized orthodontic and functional treatment planning [19,35]. Oral breathing has been linked to alterations in craniofacial morphology and upper airway function, increasing susceptibility to obstructive sleep apnea syndrome [36]. Chang and Shiao [37] demonstrated a positive correlation between mandibular plane–hyoid distance and the apnea-hypopnea index, with greater distances associated with more pronounced daytime sleepiness. In our study, individuals diagnosed with obstructive sleep apnea syndrome were excluded to isolate the effects of physiological breathing patterns. Furthermore, studies have reported positional changes in the hyoid bone after orthodontic or functional appliance therapy [38,39]. The present findings emphasize that hyoid bone position has clinical significance in orthodontic planning, particularly in identifying patients at greater risk of airway compromise. Differences in hyoid displacement should be considered in the context of obstructive sleep apnea risk and when planning orthodontic or surgical interventions aimed at improving airway stability and craniofacial balance.

In light of these findings, the clinical implications of hyoid positional differences between nasal and oral breathers merit further consideration. The inferior and more angularly favorable hyoid position observed in nasal breathers may contribute to improved airway stability, particularly by maintaining a wider pharyngeal space and reducing the risk of upper airway collapse. Conversely, the superior and less favorable positioning identified in oral breathers could predispose patients to compromised airway patency and altered craniofacial growth patterns. From a clinical standpoint, this suggests that patients—especially those with oral breathing habits—should be carefully evaluated not only for dental and skeletal discrepancies but also for potential airway vulnerability. Preventive and interceptive strategies, such as early myofunctional therapy, orthodontic interventions aimed at correcting maxillomandibular imbalances, or ENT referral for management of nasal obstruction, may help mitigate the long-term risks associated with unfavorable hyoid positioning. Moreover, in Class II skeletal patterns, where mandibular retrusion may further exacerbate airway narrowing, attention to breathing mode becomes particularly critical. Tailoring orthodontic and orthopedic treatment plans to account for both skeletal morphology and respiratory patterns may therefore enhance airway function and promote more balanced craniofacial development [40,41].

An additional noteworthy finding of this study was the significant interaction between respiration and skeletal class observed in females but not in males. This sex-specific difference may be explained by several anatomical, hormonal, and developmental factors. Anatomically, females often present with smaller upper airway dimensions and different craniofacial growth trajectories compared to males, which could amplify the influence of skeletal morphology on hyoid positioning. Hormonal influences, particularly estrogen and progesterone, are also known to affect soft tissue tone and upper airway stability, potentially modifying the relationship between breathing mode and skeletal structure in females. Developmentally, differences in pubertal growth timing and craniofacial maturation rates between genders may further account for the observed discrepancies. Taken together, these factors suggest that the interaction between respiratory mode and skeletal morphology is more pronounced in females, underscoring the importance of sex-specific considerations in both clinical assessment and treatment planning [40,41,42].

This study has several limitations that should be considered when interpreting the results. First, its retrospective and cross-sectional design limits causal inference and does not allow for evaluation of longitudinal changes in hyoid bone positioning. Second, all CBCT scans were obtained in a static seated position, which fails to capture dynamic functional activities such as swallowing, phonation, or postural adjustments—factors that can influence hyoid morphology in real-time contexts. Additionally, although the hyoid triangle technique was used to categorize breathing patterns, no objective respiratory assessments such as airflow analysis or rhinomanometry were performed, potentially introducing classification bias. The exclusion of individuals with craniofacial anomalies or obstructive sleep apnea allowed for a focus on physiological variation, but this may also limit the generalizability of findings to more clinically relevant populations. Moreover, factors such as tongue posture, head position, and neck muscle tone—known to affect hyoid location—were not evaluated, potentially confounding the observed relationships. While the study group was homogeneous in terms of gender, it consisted primarily of Class 1 patients skeletally and of those who were predominantly nasal breathers. No Class 2 oral-breathing patients were found in either gender, particularly in the 41–51 age group. Patients in the 19–29 age group constituted the majority compared to other age groups. These findings suggest that the patients presenting to the clinic were predominantly younger individuals, and the difficulty in finding a sufficient number of patients meeting the inclusion criteria in patient records of older age groups warrants a larger study group. The use of two different CBCT devices with distinct voxel sizes may have introduced variability. However, standardized protocols, calibration procedures, and consistent landmark identification minimized this potential bias. Finally, the study was conducted in a single institution with a demographically homogeneous population, which may limit its external validity across different ethnic or geographic cohorts. Future research should adopt longitudinal and multi-center designs to confirm these findings across more diverse populations. Dynamic imaging approaches could provide insights into functional changes in the hyoid during swallowing and speech. Additionally, integration of objective respiratory assessments (e.g., rhinomanometry, airflow analysis) with CBCT data may help clarify the physiological mechanisms underlying the observed differences. Exploring hormonal influences and sex-specific growth trajectories would also be valuable.

## 5. Conclusions

This study demonstrates that the position and dimensions of the hyoid bone are significantly influenced by breathing pattern, age, and skeletal morphology, with notable variations between sexes. Among these factors, breathing type consistently impacted both hyoid angle and vertical displacement, while age showed stronger associations with horizontal and vertical distances. Skeletal classification exhibited limited influence overall, with statistically significant effects observed primarily in male subgroups. These findings underscore the multifactorial nature of hyoid morphology and highlight the importance of integrating functional variables—particularly respiratory patterns—into individualized orthodontic, orthopedic, and surgical treatment planning. Accounting for age-related and sex-specific changes in hyoid position may contribute to improved airway management and craniofacial balance in clinical practice. However, further research is needed to clarify the longitudinal progression of hyoid positional changes and to assess their impact on airway function in clinical populations.

## Figures and Tables

**Table 1:** Distribution of participants by sex, age groups, breathing pattern, and skeletal pattern.

Sex	Age Group	Breathing Pattern	Class I	Class II	Class III	Total
Female	8–18 years	Oral	7	4	4	15
		Nasal	15	10	5	30
	19–29 years	Oral	16	7	13	36
		Nasal	30	19	24	73
	30–40 years	Oral	4	4	4	12
		Nasal	17	6	7	30
	41–51 years	Oral	11	4	4	19
		Nasal	18	7	13	38
	52+ years	Oral	4	0	1	5
		Nasal	20	4	6	30
Male	8–18 years	Oral	4	2	4	10
		Nasal	18	10	6	34
	19–29 years	Oral	17	8	4	29
		Nasal	28	11	23	62
	30–40 years	Oral	4	5	5	14
		Nasal	21	7	11	39
	41–51 years	Oral	5	2	2	9
		Nasal	21	8	7	36
	52+ years	Oral	2	0	2	4
		Nasal	23	7	8	38
Total			269	130	161	560

**Table 2:** Descriptive statistics of morphometric measurements.

	Mean ± Standard Deviation
Hyoid Angle (°)	88.79 ± 130.86
Hyoid Horizontal Length (mm)	14.08 ± 3.08
Hyoid Vertical Length (mm)	62.87 ± 8.14

°: Angle; mm: millimeter.

**Table 3:** Comparison of hyoid angle parameter according to age, breathing, and skeletal pattern within each gender.

Gender	Age	Breathing	Skeletal Pattern			Total		**Test Statistic**	***p***
			Class I	Class II	Class III				
Female	8–18 years	Oral	−173.77 ± 2.37	−171.34 ± 3.09	−169.37 ± 5.96	−171.95 ± 2	Age	5.411	0.320
		Nasal	161.73 ± 2.87	153.28 ± 6.68	158.3 ± 4.11	159.17 ± 2.65	Breathing	39,505.984	<0.001
		Total	60.73 ± 37.92	60.53 ± 45.44	12.67 ± 64.04	53.19 ± 26.14	Skeletal pattern	0.006	0.998
	19–29 years	Oral	−170.4 ± 1.87	−168.37 ± 3.65	−160.11 ± 3.41	−166.63 ± 1.81	Age * Breathing	2.994	0.605
		Nasal	164.15 ± 1.68	161.26 ± 2.56	158.52 ± 3.85	162.12 ± 1.49	Age * Skeletal pattern	7.323	0.600
		Total	52.31 ± 26.38	78.71 ± 32.5	48.7 ± 28.23	58.48 ± 16.5	Breathing * Skeletal pattern	2.488	0.319
	30–40 years	Oral	−167.76 ± 2.45	−168.19 ± 4.28	−170.08 ± 2.24	−168.68 ± 1.67	Age * Breathing * Skeletal pattern	17.153	0.088
		Nasal	163.23 ± 3.35	161.87 ± 4.51	149.82 ± 9.45	160.75 ± 3.04			
		Total	110.5 ± 32.37	29.85 ± 59.96	33.49 ± 54.39	72.96 ± 25.79			
	41–51 years	Oral	−167.36 ± 2.33	−170.5 ± 3.94	−167.21 ± 7.34	−167.99 ± 2.07			
		Nasal	154.92 ± 3.77	167.07 ± 3.29	159.15 ± 3.71	159.09 ± 2.21			
		Total	35.21 ± 32.86	44.32 ± 57.11	82.36 ± 38.59	53.55 ± 22.91			
	Over 52 years	Oral	−166.96 ± 3.96	−161.63 ± 3.51	−165.9 ± 2.16	−164.91 ± 1.81			
		Nasal	154.3 ± 3.22	148.38 ± 15.77	160.15 ± 3.79	154.91 ± 2.95			
		Total	109.83 ± 27.66	−6.62 ± 65.52	−2.87 ± 60.42	61.3 ± 25.64			
	Total	Oral	−169.91 ± 1.03	−168.21 ± 1.69	−164.9 ± 2.04	−168.13 ± 0.88			
		Nasal	160.76 ± 1.24	160.46 ± 2.3	158.06 ± 2.28	160.15 ± 0.99			
		Total	69.42 ± 14.09	55.07 ± 20.84	46.91 ± 18.86				
Male	8–18 years	Oral	−170.45 ± 1.2 ^A^	−173.33 ± 2.73 ^A^	−173.56 ± 1.24 ^A^	−172.4 ± 1.08 ^A^	Age	15.257	0.011
		Nasal	144.51 ± 4.12 ^B^	159.92 ± 5.57 ^B^	160.07 ± 6.18 ^B^	152 ± 3.16 ^BC^	Breathing	57,136.488	<0.001
		Total	95.75 ± 29.5	57.38 ± 49.48	38.75 ± 56.52	73.6 ± 23.68 ^ab^	Skeletal pattern	2.005	0.377
	19–29 years	Oral	−174.31 ± 1.43 ^A^	−169.13 ± 3.85 ^A^	−164.81 ± 4.81 ^A^	−169.4 ± 2.31 ^A^	Age * Breathing	20.319	0.002
		Nasal	151.8 ± 3.01 ^B^	158.19 ± 3.29 ^B^	152.91 ± 3.07 ^B^	153.6 ± 1.77 ^B^	Age * Skeletal pattern	9.977	0.359
		Total	118.15 ± 21.65	70.9 ± 43.06	114.25 ± 24.52	109.8 ± 15.53 ^a^	Breathing * Skeletal pattern	0.797	0.676
	30–40 years	Oral	−173.06 ± 2.79 ^A^	−172.03 ± 2.68 ^A^	−175.22 ± 0.97 ^A^	−173.3 ± 1.33 ^A^	Age * Breathing * Skeletal pattern	20.926	0.029
		Nasal	149.23 ± 2.41 ^B^	151.2 ± 5.34 ^B^	159.09 ± 3.28 ^B^	152.3 ± 1.93 ^B^			
		Total	106.73 ± 26.79	16.52 ± 53.48	69.94 ± 43.97	77 ± 21.96 ^ab^			
	41–51 years	Oral	−172.84 ± 1.39 ^A^	−175.41 ± 1.55 ^A^	−170.92 ± 2.5 ^A^	−172.6 ± 1.28 ^A^			
		Nasal	149.29 ± 4.48 ^B^	148.68 ± 6.12 ^B^	140.66 ± 7.57 ^B^	148.1 ± 3.24 ^BC^			
		Total	80.66 ± 34.25	19.04 ± 58.91	−41.09 ± 51.56	31.9 ± 26.57 ^b^			
	Over 52 years	Oral	−169 ± 3.03 ^A^	−169.29 ± 4.71 ^A^	−169.81 ± 1.72 ^A^	−169.4 ± 1.77 ^A^			
		Nasal	144.17 ± 3.66 ^B^	134.03 ± 7.51 ^B^	141.29 ± 6.15 ^B^	141.6 ± 2.96 ^C^			
		Total	105.34 ± 24.41	23.73 ± 51.5	37.59 ± 49.3	73 ± 21.15 ^ab^			
	Total	Oral	−172.2 ± 0.76	−172.3 ± 1.3	−171.2 ± 1.33	−171.9 ± 0.71			
		Nasal	148.2 ± 1.54	152.5 ± 2.54	152.8 ± 1.99	150.4 ± 1.11			
		Total	105 ± 11.81	44.9 ± 22.1	60.4 ± 18.88				

* Robust ANOVA, pruned mean was used as comparison method (Pruning rate 0.05), a–b: No difference between main effects with the same letter, A–C: No difference between interactions with the same letter, pruned mean ± s. Error.

**Table 4:** Comparison of hyoid horizontal length parameter according to age, breathing, and skeletal pattern within each gender.

Gender	Age	Breathing	Skeletal Pattern			Total		**Test Statistic**	***p***
			Class I	Class II	Class III				
Female	8–18 years	Oral	12.87 ± 0.57	10.18 ± 0.58	12.28 ± 0.8	12 ± 0.48	Age	17.213	0.009
		Nasal	12.96 ± 0.74	12.88 ± 0.99	10.59 ± 0.53	12.4 ± 0.51	Breathing	0.010	0.920
		Total	12.79 ± 0.44	12.11 ± 0.8	11.34 ± 0.54	12.16 ± 0.32 ^a^	Skeletal pattern	0.129	0.940
	19–29 years	Oral	12.64 ± 0.53	12.47 ± 0.82	13.27 ± 0.77	12.73 ± 0.35	Age * Breathing	2.293	0.709
		Nasal	12.09 ± 0.3	12.96 ± 0.61	13.26 ± 0.47	12.65 ± 0.25	Age * Skeletal pattern	13.773	0.175
		Total	12.25 ± 0.25	12.75 ± 0.46	13.23 ± 0.4	12.66 ± 0.2 ^ab^	Breathing * Skeletal pattern	2.114	0.369
	30–40 years	Oral	13.31 ± 1.08	14.26 ± 1.98	12.47 ± 1.08	13.35 ± 0.79	Age * Breathing * Skeletal pattern	11.316	0.290
		Nasal	14.13 ± 0.65	12.92 ± 1.06	13.78 ± 0.85	13.75 ± 0.46			
		Total	13.9 ± 0.55	13.46 ± 0.98	13.31 ± 0.67	13.62 ± 0.37 ^b^			
	41–51 years	Oral	13.22 ± 0.85	12.93 ± 0.95	14.27 ± 0.77	13.38 ± 0.55			
		Nasal	12.41 ± 0.5	12.59 ± 0.47	13.2 ± 0.98	12.53 ± 0.33			
		Total	12.67 ± 0.43	12.71 ± 0.43	13.45 ± 0.77	12.81 ± 0.28 ^ab^			
	Over 52 years	Oral	12.41 ± 1.24	14.05 ± 1.23	14.73 ± 0.8	13.81 ± 0.64			
		Nasal	13.95 ± 0.56	14.89 ± 3.06	13.26 ± 1.01	13.88 ± 0.54			
		Total	13.67 ± 0.53	14.47 ± 1.54	14 ± 0.66	13.78 ± 0.37 ^b^			
	Total	Oral	12.86 ± 0.3	12.71 ± 0.55	13.32 ± 0.37	12.98 ± 0.23			
		Nasal	12.89 ± 0.23	12.84 ± 0.36	12.95 ± 0.31	12.88 ± 0.16			
		Total	12.87 ± 0.19	12.77 ± 0.29	13.07 ± 0.23				
Male	8–18 years	Oral	11.94 ± 1.67	13.56 ± 1.06	14.55 ± 1.1	13.4 ± 0.77	Age	7.140	0.170
		Nasal	14.59 ± 0.83	14.73 ± 1.64	16.03 ± 1.75	14.8 ± 0.66	Breathing	1.940	0.170
		Total	13.9 ± 0.59	14.37 ± 1.17	15.5 ± 1.17	14.3 ± 0.51	Skeletal pattern	1.090	0.588
	19–29 years	Oral	13.6 ± 1.24	14.04 ± 1.5	18.4 ± 2.53	15.3 ± 1.21	Age * Breathing	5.190	0.312
		Nasal	15.18 ± 0.75	16.03 ± 1.17	15.16 ± 0.86	15.3 ± 0.5	Age * Skeletal pattern	15.650	0.115
		Total	14.97 ± 0.68	15.5 ± 0.95	15.67 ± 0.84	15.3 ± 0.46	Breathing * Skeletal pattern	1.910	0.398
	30–40 years	Oral	15.88 ± 1.79	15.89 ± 0.95	15.65 ± 0.77	15.8 ± 0.64	Age * Breathing * Skeletal pattern	8.900	0.455
		Nasal	15.76 ± 0.71	14.64 ± 0.89	15.62 ± 1.26	15.5 ± 0.57			
		Total	15.78 ± 0.65	15.16 ± 0.66	15.63 ± 0.93	15.5 ± 0.41			
	41–51 years	Oral	13.93 ± 1.13	14.04 ± 1.82	15.96 ± 1.14	14.8 ± 0.76			
		Nasal	14.93 ± 0.73	14.94 ± 1.75	13.98 ± 1.2	14.7 ± 0.58			
		Total	14.58 ± 0.57	14.58 ± 1.22	15.14 ± 0.86	14.7 ± 0.45			
	Over 52 years	Oral	14.82 ± 0.71	14.4 ± 1.05	11.6 ± 0.68	13.6 ± 0.65			
		Nasal	15.66 ± 0.79	15.42 ± 1.66	14.46 ± 0.98	15.4 ± 0.6			
		Total	15.52 ± 0.68	15.05 ± 1.09	13.51 ± 0.81	14.8 ± 0.48			
	Total	Oral	14 ± 0.55	14.5 ± 0.5	15.1 ± 0.63	14.5 ± 0.33			
		Nasal	15.2 ± 0.34	15 ± 0.54	15.1 ± 0.52	15.1 ± 0.25			
		Total	15 ± 0.3	14.8 ± 0.38	15.1 ± 0.42				

* Robust ANOVA pruned mean was used as comparison method (Pruning rate 0.05). a–b: No difference between main effects with the same letter. pruned mean ± s. Error.

**Table 5:** Comparison of hyoid vertical height parameter according to age, breathing, and skeletal pattern within each gender.

Gender	Age	Breathing	Skeletal Pattern			Total		**Test Statistic**	***p***
			Class I	Class II	Class III				
Female	8–18 years	Oral	55.06 ± 1.05	54.79 ± 1.61	58.3 ± 3.88	55.85 ± 1.19	Age	38.846	<0.001
		Nasal	55.6 ± 1.49	57.99 ± 2.19	60.3 ± 2.69	57.23 ± 1.1	Breathing	52.214	<0.001
		Total	55.63 ± 0.99	57.08 ± 1.66	59.42 ± 2.16	56.7 ± 0.77 ^a^	Skeletal pattern	0.112	0.947
	19–29 years	Oral	54.14 ± 1.16	52.5 ± 0.7	51.82 ± 1.47	52.94 ± 0.73	Age * Breathing	6.299	0.232
		Nasal	57.73 ± 0.98	58.42 ± 1.2	59.36 ± 1.44	58.44 ± 0.69	Age * Skeletal pattern	9.914	0.388
		Total	56.44 ± 0.77	56.88 ± 1.01	56.6 ± 1.26	56.53 ± 0.58 ^a^	Breathing * Skeletal pattern	8.225	0.029
	30–40 years	Oral	62.53 ± 0.77	52.71 ± 1.83	58.14 ± 1.47	57.79 ± 1.54	Age * Breathing * Skeletal pattern	15.648	0.122
		Nasal	59.55 ± 1.06	63.87 ± 2.44	62.96 ± 3.63	61.13 ± 1.13			
		Total	60.34 ± 0.85	59.4 ± 2.55	61.2 ± 2.44	60.12 ± 0.92 ^b^			
	41–51 years	Oral	57.19 ± 1.4	57.59 ± 2.54	55.15 ± 2.16	56.84 ± 1.04			
		Nasal	62.29 ± 1.67	62.66 ± 1.55	62.83 ± 2.03	62.49 ± 1.05			
		Total	60.26 ± 1.2	60.82 ± 1.54	61.02 ± 1.84	60.52 ± 0.84 ^b^			
	Over 52 years	Oral	56.43 ± 2.39	58.02 ± 1.47	51.29 ± 0.74	54.94 ± 1.26			
		Nasal	64 ± 0.97	63.72 ± 5.68	62.37 ± 3.35	63.77 ± 1.14			
		Total	62.81 ± 1.07	60.87 ± 2.97	56.83 ± 2.61	60.91 ± 1.07 ^b^			
	Total	Oral	56.14 ± 0.69 ^A^	54.77 ± 0.79 ^A^	53.66 ± 0.91 ^A^	55.03 ± 0.47			
		Nasal	59.83 ± 0.59 ^B^	60.06 ± 0.9 ^B^	61.06 ± 0.99 ^B^	60.19 ± 0.44			
		Total	58.65 ± 0.48	58.15 ± 0.71	58.34 ± 0.81				
Male	8–18 years	Oral	51.76 ± 2.64	54.54 ± 1.04	57.99 ± 2.49	54.8 ± 1.42	Age	84.460	<0.001
		Nasal	65.95 ± 2.19	58.2 ± 3.7	61.26 ± 2.5	63 ± 1.66	Breathing	72.180	<0.001
		Total	63.47 ± 2.21	57.08 ± 2.59	60.07 ± 1.84	60.7 ± 1.39 ^a^	Skeletal pattern	10.850	0.008
	19–29 years	Oral	62.59 ± 0.82	62.83 ± 2.68	62.15 ± 3.79	62.5 ± 1.43	Age * Breathing	8.650	0.109
		Nasal	67.38 ± 0.89	69.27 ± 1.88	68.02 ± 1.44	68 ± 0.67	Age * Skeletal pattern	15.740	0.127
		Total	66.74 ± 0.85	67.55 ± 1.72	67.08 ± 1.41	67.1 ± 0.67 ^b^	Breathing * Skeletal pattern	1.280	0.536
	30–40 years	Oral	65.07 ± 3.42	68.46 ± 1	63.92 ± 0.81	66 ± 1.22	Age * Breathing * Skeletal pattern	10.270	0.373
		Nasal	69.03 ± 0.93	72.86 ± 1.8	66.75 ± 1.88	69.2 ± 0.88			
		Total	68.57 ± 0.89	71.03 ± 1.31	66 ± 1.43	68.4 ± 0.72 ^bc^			
	41–51 years	Oral	64.95 ± 2.34	66.03 ± 2.27	62.14 ± 1.5	64 ± 1.16			
		Nasal	70.7 ± 1.14	73.81 ± 1.58	66.53 ± 4.08	71 ± 0.84			
		Total	69.39 ± 1.06	70.7 ± 1.87	63.97 ± 1.94	68.3 ± 0.91 ^bc^			
	Over 52 years	Oral	66.23 ± 3.22	64.93 ± 2.5	59.26 ± 3.53	63.5 ± 1.92			
		Nasal	73.39 ± 1.07	80.32 ± 3.74	69.75 ± 2.3	73.6 ± 1.17			
		Total	72.41 ± 1.09	74.72 ± 3.58	66.26 ± 2.48	71.2 ± 1.08 ^c^			
	Total	Oral	62.5 ± 1.57	63.8 ± 1.35	61.2 ± 0.95	62.5 ± 0.75			
		Nasal	69.5 ± 0.55	70.2 ± 1.52	67.1 ± 0.9	69 ± 0.47			
		Total	68.4 ± 0.56 ^a^	67.8 ± 1.11 ^ab^	65.3 ± 0.78 ^b^				

* Robust ANOVA pruned mean was used as comparison method (Pruning rate 0.05). a–c: No difference between main effects with the same letter. pruned mean ± s. Error.

## Data Availability

The datasets generated and/or analyzed during the current study are not publicly available due to institutional data privacy policies but are available from the corresponding author on reasonable request.

## Abbreviations

The following abbreviations are used in this manuscript:

CBCT	Cone Beam Computed Tomography
ICC	Intraclass Correlation Coefficient
RGn	Retrognathion
PL	Palatal Plane
C3	Third Cervical Vertebra
ANB	A–Nasion–B Angle
SNA	Sella-Nasion-A Angle
SNB	Sella-Nasion-B Angle
